# Depression, anxiety, and personal recovery outcomes after group vs individual transdiagnostic therapy: a brief report

**DOI:** 10.1038/s41598-024-55093-7

**Published:** 2024-02-28

**Authors:** Sayani Paul, Lynn Zhu, Jane Mizevich, Lindsay Slater

**Affiliations:** 1 Ontario Shores Centre for Mental Health Sciences, 700 Gordon St, Whitby, ON L1N 5S9 Canada; 2 Faculty of Health Sciences, Ontario Tech University, Oshawa, Canada

**Keywords:** CBT, Unified protocol, Personal recovery, Transdiagnostic, Anxiety, Depression, Health care, Health services

## Abstract

Unified Protocol for Transdiagnostic Treatment of Emotional Disorders (UP) is an evidence-informed treatment utilizing Cognitive Behavioural Therapy (CBT) treatment principles. UP has demonstrated promising treatment effects comparable to single disorder protocol across several mental disorders. Its impact on personal recovery in anxiety and depression has not been examined. This study compares clinical and personal recovery outcomes of UP treatment for depression and anxiety disorders when delivered in a group vs. individual format. Retrospective chart review of outcomes was conducted for outpatients receiving 12-week individual (*n* = 65) and group (*n* = 62) UP treatment in a specialized psychiatric hospital. Descriptive and repeated measures ANOVA analyses were conducted on outcomes on Overall Depression Severity and Impairment Scale, Overall Anxiety Severity and Impairment Scale, Recovery Assessment Scale administered pre and post treatment. On average, participants in both group and individual UP treatment showed improvements in anxiety, depression, and recovery scores. Greater proportion of group participants showed improvements on two interpersonal-focused domains of personal recovery. Results indicate group UP treatment is comparably effective compared to individual UP in improving clinical and recovery outcomes, and treatment modality affects the degree of personal recovery. Overall findings offer important clinical promise of UP treatment as a transdiagnostic treatment option for individuals with anxiety and depression.

## Introduction

Globally, depressive and anxiety disorders are leading contributors of health-related burden on quality of life^1^. Effectiveness of Cognitive Behavioural Therapy (CBT) is well documented^2^ as a gold standard for treatment of mood and anxiety disorders ^3^. However, comorbidities of other complex mental health disorders can complicate and decrease the effectiveness of traditional single disorder protocol (SDP)^4^. Further, it may be costly to provide diagnosis specific treatment, especially in an outpatient setting.

To effectively deal with comorbidities and facilitate access to treatment, transdiagnostic interventions have been developed^4^, which apply the same underlying treatment principles across different mental disorders without tailoring the protocol to each diagnosis^5^. Unified Protocol for Transdiagnostic Treatment of Emotional Disorders (UP)^6^ is an evidence-informed psychotherapy that when delivered individually^7–9^, has demonstrated promising treatment effects compared to SDP^7^. Few studies indicate that when delivered in a group setting, UP can be as effective as individual UP^4,10,11^. This is in line with a strong body of research indicating that group psychotherapy demonstrates outcomes equivalent to those of individual therapy^12^. The authors found that group psychotherapy demonstrated large effects of disorder-specific symptom reduction associated with anxiety, obsessive compulsive disorder and depression; medium effects for eating disorders and posttraumatic stress disorder, and small effects for substance use disorders and schizophrenia. They also found that the differences between group psychotherapy and other active conditions such as individual therapy are negligible in terms of effect size^12^.

Personal recovery is an ongoing individual process that focuses on instilling a sense of purpose and hope, strengthening connections, making meaning of individual’s experiences of mental illness and empowering individuals to re(establish) a meaningful life despite persistent symptoms^13,14^. Recovery in the mental health context refers to the process of changing one's attitudes, values, feelings, goals, and skills to live a satisfying life within the limitations caused by illness. It contrasts with the clinical recovery concept that emphasizes one’s psychiatric symptoms and functioning^15^. The concept of personal recovery aligns well with positive psychiatry and positive psychology as they imply optimism that despite the mental illness symptoms, improvement is possible, and well-being is achievable^16^. Aligning with positive psychiatry that encompasses psychological aspects such as optimism, resilience, personal mastery, coping, self-efficacy, social engagement, and spirituality, personal recovery involves a healing process and restoring balance despite the mental illness^13,16,17^. In this process the individual learns to accept the mental illness and shifts their attitude towards optimism and personal and professional goal attainments. Research is limited on impact of UP on personal recovery outcomes. A few studies have examined the impact of single disorder CBT on personal recovery in schizophrenia^18,19^, but we did not find any studies focusing on UP or other transdiagnostic interventions and personal recovery.

Research shows that the therapeutic benefits of group psychotherapy, in general include development of social techniques, imitative behaviours, interpersonal learning and group cohesiveness^19^, all of which are in line with personal recovery principles. Additionally, Barlow and Farchione^20^ highlighted that group psychotherapy facilitates normalization of experiences from listening to others with similar difficulties, provides opportunities for exposures, promotes engagement because of group support or from watching others, as well as sometimes makes it easier to apply treatment concepts and skills by applying it to a group member’s situation, which is also consistent with recovery principles. In this way, offering UP in a group setting may more strongly support principles of the recovery model than individual UP treatment and therefore show greater recovery-related outcomes for group therapy participants than for individual therapy participants.

It has been noted that group psychotherapy helps to increase the availability of resources by having one practitioner deliver treatment to a group of individuals^20^. An especially cost-effective way of delivering group psychotherapy is by using transdiagnostic interventions, which can target multiple diagnoses within the same group. Transdiagnostic interventions such as UP align well with the stepped care approach to treatment^21^. According to the Mental Health Commission of Canada^22^ stepped care is a person-centred approach to mental health care that organizes and delivers evidence-based programming aligned with recovery principles and improves equitable and timely access to mental health resources. Since the rationale behind stepped care is to provide more cost-effective and less time-intensive care^23^, transdiagnostic approaches such as UP are very much in line with this philosophy in that they can address more than one disorder at a time without needing to provide different protocols for each disorder.

This study compares clinical and personal recovery outcomes of UP treatment for depression and anxiety disorders when delivered in a group vs. individual format. Our hypotheses were:

1. Participants in both group and individual setting will have similar clinical outcomes.
2. Participants in group UP may experience better personal recovery outcomes compared to those who received UP individual session.

## Methods

### Participants and setting

This study was conducted at the Anxiety and Mood Disorders (AMD) clinic—an outpatient program at Ontario Shores Centre for Mental Health Sciences (Ontario Shores)—a public tertiary psychiatric teaching hospital located in Ontario, Canada’s most populous province. As a leader in recovery oriented and client centered care, the hospital offers a range of specialized mental health services to individuals struggling with mental illness and their families.

Individuals who received a DSM-5 diagnosis of anxiety and/or mood disorders after a standard diagnostic assessment based on a clinical interview with a psychiatrist or a nurse practitioner at Ontario Shores were referred to the AMD clinic where they could attend either individual or group psychotherapy, based on their preference. In this study, we included adults (18–65 years) with moderate to severe anxiety disorders and/or depression who received individual (*n* = 65) and group (*n* = 62) UP treatment. Consistent with the transdiagnostic goals of unified CBT, participants in both group and individual therapy presented with a variety of chronic and severe mental health primary diagnoses. Individuals with acute and active psychosis, significant cognitive impairment, and those with a tendency to be disruptive in groups were excluded. This study was approved by the Research Ethics Board (REB) of Ontario Shores (REB # 18-018-B).

### Study design

We reviewed the clinical charts of eligible participants to compare measures captured pre- and post-UP among participants receiving individual and group UP treatment. Participants receiving group and individual UP were selected and matched as closely as possible on potential confounders, i.e., gender, age, diagnosis, symptom severity, and number of sessions attended.

### UP treatment

Between August 2018 to March 2020, individuals participated in a 12-week UP therapy, either in group or individual format, co-facilitated by trained psychotherapists following the guidelines of their professional regulatory colleges. Each group UP session was 90 min and attended by 6 to 12 participants weekly, while individual UP was conducted at therapist’s office for 50 min every week. Treatment content followed the UP manual^7^ covering nine modules (Appendix A) and were same for individuals and groups. Practice homework and workbook readings were assigned after each individual and group UP session. Group UP also offered participants the opportunity to practice the skills learned in group or pairs.

### Data collection

All participants completed the following instruments before and at the completion of the UP treatment with a psychotherapist.

1. Overall Anxiety Severity and Impairment Scale (OASIS)—a five-item self-report measure that is used to assess severity and impairment associated with any anxiety disorder or multiple anxiety disorders^24^. It is scored out of 20 and present with strong psychometric properties, indicating the applicability of this scale in a wide range of anxiety related disorders. Higher OASIS scores specify greater anxiety-related severity and impairment.
2. Overall Depression Severity and Impairment Scale (ODSIS) is a brief, five-item scale for assessing the frequency and intensity of depressive symptoms, as well as functional impairments in pleasurable activities, occupational, and interpersonal relationships due to depression^25^. It is also scored out of 20 and higher ODSIS indicates greater depression severity and impairment. The validity and reliability of the scale is well established in multiple cultures^26^.
3. Recovery Assessment Scale (RAS-24 [RAS]) is a self-reflective assessment used to measure individuals’ perceptions of individual recovery^27^. The soring is done on a five-point Likert scale, with higher score indicating better personal recovery. RAS is one of the most widely used personal recovery measures^28^ in recovery-oriented mental healthcare settings. Its 24 items have shown good reliability, validity, and utility^29^, and represent five domains—personal confidence and hope, willingness to ask for help, goal and success orientation, reliance on others, and no domination by symptoms.

Ontario Shores REB approved the study as a minimal risk retrospective chart review with no additional patient interaction. Obtaining informed consent would have required contacting each patient directly post-therapy, increasing the risks to patient privacy and burden. To protect patient privacy, the researchers were blinded to identifying patient information during the chart review, and only anonymized study data were extracted and analysed. The inclusion of data for this research did not impact patient therapy in any way. All research procedures have been conducted in accordance with the Declaration of Helsinki.

### Data analysis

We conducted repeated measures ANOVA analysis (with a Bonferroni correction at *p* = 0.0125 for multiple comparisons) on pre and post outcome measures. To understand frequency of change, we summarized the score changes descriptively. Due to the relatively small sample and heterogeneity in participant comorbidities, we are underpowered to complete subgroup analyses.

## Results

**Figure 1:**
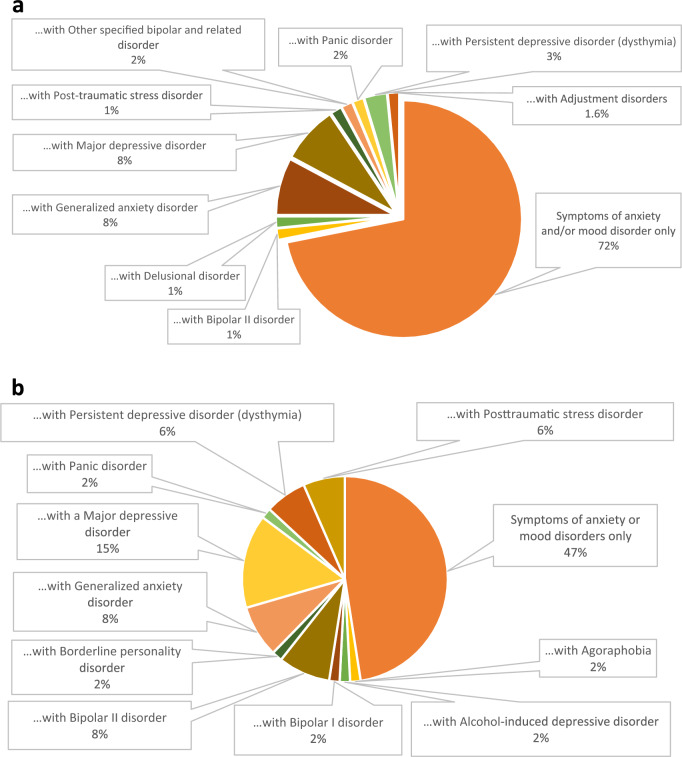
(**a**) Comorbidity distribution of *group* Unified Protocol CBT participants showing the transdiagnostic profiles of the program attendees. All participants were receiving services at an Anxiety and Mood Disorders clinic, and presented with symptoms of anxiety and/or mood disorders with or without an additional mental health disorder. (**b**) Comorbidity distribution of *individual* Unified Protocol CBT participants showing the transdiagnostic profiles of the program attendees. All participants were receiving services at an Anxiety and Mood Disorders clinic and presented with symptoms of anxiety and/or mood disorders with or without an additional mental health disorder.

One hundred and twenty-five participants took part in UP treatment, with 64 participants opting for group UP treatment (62.5% female; 44.5 ± 10.4 years [25–65]) and 61 participants in the individual UP treatment (59.0% female; 44.0 ± 10.8 years [25–65]). All participants presented with anxiety and/or a mood disorder, with 28.1% of group and 52.5% of individual UP treatment participants also presenting with additional complex mental health diagnoses (Fig. 1a,b). Table 1 shows statistically significant improvements in anxiety, depression and overall recovery scores among participants who completed *either* the individual or the group UP. On average, participants of both group and individual UP treatment showed improvements in scores measuring anxiety (OASIS; group: 15.81% improvement in scores post-intervention, *p* < 0.001; individual: 16.6%,* p* = 0.015), depression (ODSIS; group: 19.98% improvement, *p* = 0.004; individual: 21.91%, *p* < 0.001), and recovery (RAS; group: 9.10% improvement, *p* = 0.002; individual: 9.40%, *p* < 0.001). Statistically significant differences between individual and group change scores were not found. Table 1 suggests greater proportion of group UP participants showed improvements in their ‘Willingness to ask for help’ (35.2% of group participants vs 21.8% individual) and ‘Reliance on others’ subscales of the RAS (28.07% group participants vs 24.1% individual).

**Table 1:** (a) Clinical and personal recovery outcomes of participants before and after attending either individual or group UP, (b) Greater proportion of participants improved on interpersonal-based personal recovery subscales after group UP vs individual (box).

(a)	Individual			Group		
	Mean scores pre-UP	Mean scores post-UP	p*	Mean scores pre-UP	Mean scores post-UP	P*
Anxiety (OASIS)	11.79 ± 4.36	9.83 ± 4.18	< 0.001*	11.51 ± 4.17	9.69 ± 5.13	0.015
Depression (ODSIS)	11.82 ± 4.21	9.23 ± 4.87	< 0.001*	11.11 ± 4.61	8.89 ± 5.28	0.004*
Recovery (RAS)	73.73 ± 12.41	80.66 ± 16.46	0.002*	74.98 ± 10.75	81.80 ± 15.67	< 0.001*
(b)						


*Bonferroni corrected *p* < 0.0125 was used for significance testing.

## Discussion and conclusion

Aligning with previous research^4,10,11^ and our first hypothesis, we found that group UP is comparably effective in improving clinical outcomes compared to individual UP. We also found greater improvements in some recovery outcomes post-group UP, suggesting promising advantages of group modality over individual treatment. Group UP offered opportunities for participants to practice asking one another for help, and may add additional layers of peer support, exposure, and shared learning environment that enhanced personal recovery outcomes related to interpersonal trust. Although the need for recovery-focused CBT is well documented^19^, research have not yet focused on the impact of UP on personal recovery in anxiety and depression. Our results suggest UP is associated with more consistent effects for anxiety and depression than for personal recovery. This is to be expected as participants were primarily seeking treatment for anxiety and mood disorder symptoms. As well, personal recovery is a highly personal construct with multiple domains and is dependent on a variety of influences, as demonstrated by the differences shown in participants’ recovery subscale outcomes. This highlights the importance of prioritizing individual recovery subscale scores, as well as the overall scores. Overall, these findings expand the literature showing some advantages of group UP over individual treatment to promote recovery-oriented care for individuals with comorbid conditions. It is worthy to note that compared to the individual UP group, a smaller proportion of group UP participants presented with another mental health disorder additional to anxiety and/or depression. This suggest the promising effects of group UP on clinical and recovery scores may also be due to lower complexity patients self-selecting for this type of therapy modality.

Our findings offer important clinical and research implications. Given the high demand for mental health services worldwide, group UP can increase simultaneous access to evidence-informed psychotherapy by multiple clients, thereby reducing wait times and strains on the mental health system. Further, given the considerably high (75%) comorbidity for depressive and anxiety disorders^30^, many individuals do not fit into a specific SDP. Beyond the Unified Protocol’s potential to offer treatment for those with comorbidities, this therapy approach is also promising to treat clients across multiple diagnoses together (e.g. post-stress disorder, bipolar disorders, etc.). The transdiagnostic nature of the UP program is captured in the Fig. 1a and b. Such individuals may benefit from UP, since transdiagnostic CBT are more effective in addressing co-morbidity than SDP CBT^31^. Additionally, UP involves simplified training efforts requiring fewer hours to train clinicians, this treatment modality is more cost-effective^7^ and at times preferred by clinicians who find SDP overwhelming to learn and implement effectively, especially when psychiatric comorbidity is present.

Improvements post-group UP in interpersonal-related personal recovery outcomes may be promising to promote sustained treatment effectiveness. However, further exploration of the clinical mechanisms and dynamics that group UP offers is required. UP modules include interventions not offered in all SDPs (e.g., mindfulness, emotion regulation, motivational interviewing), and thus future studies are needed to explore if and how these components advance personal recovery outcomes. More research is also needed to explore the efficacy of this transdiagnostic intervention in other populations, such as children and adolescents, as well as when UP is delivered virtually.

Study limitations include the relatively small sample size limiting subgroup analysis within treatment modalities. As well, this study was not able to provide insights about the sustainability of the effects observed. Given that client contact post-discharge was not usual protocol, the Research Ethics Board did not approve further research-related follow up to limit client burden. Further, these findings may not be applicable to inpatient populations who may have more complex needs.

Despite these limitations, this study offers important clinical promise for individuals with anxiety and depression and expands the current literature by demonstrating that group UP is comparable to individual UP, and present with advantageous over individual UP on select personal recovery outcomes. The findings also show the promise of UP as an additional evidence-based psychotherapy option that can benefit individuals with comorbid or more complex mental health disorders.

### Supplementary Information

Supplementary Information.

## Data Availability

The datasets generated and/or analysed during the current study are not publicly available due to privacy reason but are available from the corresponding author on reasonable request.
