# Supplementary material for: Depression, anxiety, and personal recovery outcomes after group vs individual transdiagnostic therapy: a brief report
Source: Sci Rep. 2024 Feb 28;14:4855. doi: 10.1038/s41598-024-55093-7 (PMC10901779; doi:10.1038/s41598-024-55093-7)
Supplement: Supplementary file 1 — Supplementary Information. [file 41598_2024_55093_MOESM1_ESM.docx]

**Appendix A**

**Unified Protocol for Transdiagnostic Treatment of Emotional Disorders (UP)**

Module 1: What are Emotional Disorders?

Module 2: Setting Goals for Treatment and Maintaining Motivation

Module 3: Understanding Your Emotions

Module 4: Nonjudgmental Present Focused Awareness

Module 5: Understanding Thoughts: Jumping to Conclusions and Thinking the Worst

Module 6: Understanding Emotional Behaviours

Module 7: Understanding and Confronting Physical Sensations

Module 8: Putting it into Practice

Module 9: Moving On From Here – Recognizing Your Accomplishments
